# Hepatitis C viral load and genotypes among Nigerian subjects with chronic infection and implication for patient management: a retrospective review of data

**DOI:** 10.11604/pamj.2020.37.335.20299

**Published:** 2020-12-10

**Authors:** Rosemary Ajuma Audu, Azuka Patrick Okwuraiwe, Fehintola Anthonia Ige, Olufunke Oluwatosin Adeleye, Charles Asabamaka Onyekwere, Olufunmilayo Adenike Lesi

**Affiliations:** 1 Center for Human Virology and Genomics, Nigerian Institute of Medical Research, Lagos, Nigeria,; 2 Department of Medicine, College of Medicine, Olabisi Onabanjo University Teaching Hospital, Lagos, Nigeria,; 3 Department of Medicine, College of Medicine, Lagos State University Teaching Hospital, Lagos, Nigeria,; 4 Department of Medicine, College of Medicine, University of Lagos, Lagos, Nigeria

**Keywords:** Hepatitis C virus, viral load, genotype, management, Nigeria

## Abstract

**Introduction:**

Hepatitis C Virus (HCV) is highly infectious with no currently available vaccine. Prior to treatment, it is recommended to confirm HCV infection with either quantitative or qualitative nucleic acid test. Access to these assays in Nigeria is limited but for effective management of patients, HCV viral load (VL) prior to therapy is required and genotype may be needed in some instances. This study aimed at reviewing the pattern of HCV viral load and genotype in the country, and its implication in patient management.

**Methods:**

this was a retrospective study that involved data abstraction from an electronic database of an accredited laboratory between June 2013 and May 2017. De-linked data were abstracted from records of adult subjects with HCV VL and genotype results, these were analysed using Microsoft Excel 2010 and SPSS v20.

**Results:**

within the study period, 346 subjects had baseline VL and 134 (38.7%) had genotype results available. Of these, 202/346 (58.4%) had detectable VL results with higher prevalence in males (64.7%) and ≥51years (42.5%) age group. The median VL among 202 subjects was 407,430 (IQR: 96,388 - 1,357,012) IU/mL. Distribution of genotypes showed that genotypes 1 and 4 had prevalence of 63.2% and 16.8% respectively.

**Conclusion:**

genotypes 1 and 4 have the highest prevalence. A greater proportion of subjects had VL values ≤800,000 IU/mL, an indication that they are more likely to respond well to available antiviral therapy hence, access to these antivirals will greatly improve management of HCV infection in Nigeria.

## Introduction

Hepatitis C virus (HCV) infection is caused by a blood borne virus which is 10 times more infectious than HIV with no currently available vaccine [1]. Globally, about 110 million persons have been estimated to have a history of HCV infection while 80 million have chronic infections [2]. The sub-Saharan African region has been reported to have moderate prevalence ranging from 1.5 - 3.5% [2]. In Nigeria, the prevalence of HCV is 2.2% [3]. HCV is a small, positive single stranded, RNA-enveloped virus with seven major genotypes and sixty-seven subtypes [4]. Identification of HCV RNA confirms active HCV infection and is a pre-requisite for treatment. HCV pre-treatment VL is an important prognostic marker and predictive sign for the outcome of antiviral therapy. The distribution of the genotypes varies according to geographical locations. Until recently, access to HCV VL and genotype testing in Nigeria was limited only to the Center for Human Virology and Genomics at the Nigerian Institute of Medical Research, Lagos. It was only in 2017, when the drive to scale up access to HCV treatment was initiated that the capacity of a few other laboratories was strengthened to assay for HCV VL alone. During the period under review, the cost of HCV VL was USD130 while the genotype was about USD65. This obviously limited access to these tests hence, the need for the current efforts to intensify access to care. Through the efforts of Clinton Health Access Initiative, the cost of HCV VL has declined to USD55 and efforts are still in place to further reduce it to ensure improved access to treatment. The cost of the test was relatively high in the past years. Out-of pocket patient cost was the major means of paying for the assays hence, not many subjects could access the tests.

Genotype 1 is prevalent in USA, Europe, Japan and West Africa; genotype 4 in Egypt and Central Africa while genotypes 3 and 6 are prevalent in Asia [5-7]. In Nigeria, with the exception of genotype 5, all other genotypes have been identified with genotype 1 being the most prevalent [8]. Until recently, it was necessary to determine the genotype of the infecting virus before commencing treatment with peg-IFN and ribavirin because the dose and duration of treatment varied depending on the genotype identified in an individual [9]. The treatment duration for genotypes 1 and 4 was 48 weeks, while genotypes 2 and 3 were treated for 24 weeks [10]. Currently, direct-acting antivirals (DAAs) are used for treatment of HCV with >90% cure rate, and the 2016 hepatitis treatment guidelines provide recommendations as to the preferred and alternative drugs [10] however; there is still some variation in recommended regimen. Therefore, there has been a need to know the genotypes for effective treatment options. However, determination of genotypes may no longer be necessary as the pangenotypic drugs become more readily available and accessible. Although use of newer DAAs has transformed the treatment of HCV infection globally, access to these drugs is just being pioneered in Nigeria. Therefore, knowledge of pre-treatment viral load (VL) and genotype remain relevant in determining treatment success in certain clinical situations using the more available older DAAs and pegylated interferon therapy. HCV VL is important in monitoring subjects on therapy as several studies have shown that subjects with high baseline values ≥800,000 RNA copies/mL do not respond as well as subjects with lower VLs [11-13] on standard interferon treatment. Rapid virological response (that is, undetectable VL at 4weeks) is a prognostic tool for predicting treatment response. Due to limited studies on HCV VL and genotype in Nigeria, this study aimed at reviewing data generated from a hepatitis reference laboratory to determine the pattern of HCV VL and genotype in the country, and its implication in patient management.

## Methods

**Study design and population:** this is a retrospective study that involved data abstraction and analysis from an electronic database, File Maker Pro version 12. The study population consisted of subjects previously screened positive for HCV who had visited the Center for Human Virology and Genomics at the Nigerian Institute of Medical Research, Lagos for HCV VL estimation and genotype determination, between June 2013 and May 2017.

**Data abstraction and inclusion criteria:** data were abstracted from records of adult subjects ≥18 years who had at least baseline HCV VL result. Some subjects had both VL and genotype results. Patient data with only genotype results without baseline VL results were excluded. Data abstracted were de-linked and cleaned before analysis.

**Ethical considerations:** ethical approval was obtained for this study from the Institutional Review Board of the Nigerian Institute of Medical Research.

**HCV quantitative test:** the HCV VL was estimated using the HCV Quantitative Test, v.2.0 test kits (Roche, Germany) on the COBAS AmpliPrep instruments, TaqMan48 and 96 analyzers. There are three major steps which are all automated and include: specimen preparation, reverse transcription, and simultaneous polymerase chain reaction (PCR) amplification and detection of target RNA. The limit of detection of the assay is 15 - 100,000,000IU/mL.

**HCV genotyping:** the linear array HCV genotyping test (Roche, Germany) was used for determining the genotype. The test is based on five major processes namely: specimen preparation; reverse transcription; PCR amplification; hybridization; and detection of the probe-bound amplified products by colorimetric determination.

**Statistical analysis:** the abstracted data were analysed using Microsoft Excel 2010. Summary statistics including mean, median, frequencies, percentages and rates were computed. Statistical Package for the Social Sciences (SPSS) v20 was used to test assumptions. Kruskal-Wallis test and correlation coefficient were used to determine association and strength of relationship between the VL and genotypes.

## Results

Within the time span of four years, 457 records of HCV VL from subjects aged ≥18years (ranging from 18 - 89 years) were abstracted from the database. Of this, 346 (75.7%) had baseline VL result available in the database, 23/346 (6.6%) had multiple (ranging from 2-7) VL results and 134 (38.7%) had genotype results in addition to VL. The male to female ratio was 2.1: 1 and the median age was 49 years. Ten subjects had missing records of sex.

**Distribution of HCV RNA viral load:** out of a total of 346 subjects with available baseline VL results, 144 (41.6%) had undetectable VL results while 202 (58.4%) had detectable results. The median VL among the 202 subjects was 407,430 (IQR: 96,388 - 1,357,012) IU/mL. A total of 123/202 (60.9%) subjects had VL values between 15 - ≤800,000 IU/mL while 79/202 (39.1%) had VL values ≥800,000 IU/mL. Table 1 shows the age and sex distribution of study subjects with detectable VL. There were more males (64.7%) with detectable VL than females (30.9%). Similarly, detectable VL was found more in those within the age groups of ≥51 (42.5%) then 41-50 (24.6%) years of age.

**Table 1:** age and sex distribution of study subjects with detectable HCV viral load

Age group (years)	n (%)	Male (%)	Female (%)	Unknown (%)
≤30	42 (12.1)	27	14	1
31-40	72 (20.8)	46	22	4
41-50	85 (24.6)	52	30	3
≥51	147 (42.5)	99	41	7
**Total**	**346**	**224 (64.7)**	**107 (30.9)**	**17 (4.3)**

**Distribution of genotypes:** out of 134 samples assayed for genotyping, 39 (29.1%) had undetected results because of the absence of detectable HCV RNA in the samples. Distribution of genotypes for 95 samples with detectable VL values showed that genotype 1 had the highest prevalence 60/95 (63.2%) (Table 2). Genotypes 4 and 3 were next with prevalence of 16.8% and 10.5% respectively. Genotype 2 had the lowest prevalence of 7.4%, while the mixed genotypes accounted for 2.1%. However, only genotype 2 had a median viral load value greater than 800,000 IU/mL, even though, all genotypes had upper quartile VL values greater than 800,000 IU/mL. Nonetheless, there was no significant (p<0.05) relationship between genotype and VL results obtained. The distribution of HCV median VL in each gender by genotype is shown on Table 3. The median VL values were not significantly (p<0.05) associated with any sex across all genotypes, though, the highest VL value of 1,112,436 IU/mL for genotype 2 was observed in a female patient.

**Table 2:** distribution of HCV genotypes by detectable viral load values

Genotype	n (%)	HCV viral load (IU/mL)	
		*Median	Interquartile range
1	60 (63.2)	597,272	104,337 - 1,592,901
2	7 (7.4)	874,000	156,000 - 1,399,322
3	10(10.5)	511,664	170,223 - 6,295,605
4	16 (16.8)	343,567	47,050 - 924,150
Mixed Genotypes	2 (2.1)	Nil	Nil

* There is no significant (p < 0.05) relationship between genotype and VL (H = 3.3085)

**Table 3:** distribution of HCV median viral load in each gender by genotype

Genotype	N	*Female			*Male		
		n	Median age	#Median VL (IU/mL)	n	Median age	#Median VL (IU/mL)
1	55	13	48	778,522	42	48.5	597,272
2	7	1	58	1,112,436	6	42.5	533,787
3	9	3	48	296,823	6	45.5	511,664
4	15	2	42	40,948.5	13	53	546,785

* Sex was not indicated for 7 subjects; #There is no significant difference (p<0.05) in VL between males and females in relation to the genotype, H = 0.0214.

## Discussion

The national guideline for the prevention, care and treatment of viral hepatitis in Nigeria [14], requires a confirmatory test with HCV RNA after a positive antibody serology before commencement of treatment and the confirmation of sustained virological response 12 weeks after completion of treatment so that patients who do not achieve sustained virological response are evaluated for re-treatment; Until recently, it was necessary to determine the genotype of the infecting virus before commencing treatment with peg-IFN and ribavirin because the dose and duration of treatment varied depending on the genotype identified in an individual, thus this study reviewed the prevalence of HCV VL and genotype in this environment. In the current study, HCV viraemia was identified in 58.4% of the samples reviewed. The absence of viraemia in the presence of anti-HCV antibodies may suggest resolution of HCV infection in 41.6% of the subjects. This is consistent with current evidence that suggests that 15 - 45% of acutely infected individuals, spontaneously clear the virus from their system within six months of infection, even in the absence of treatment [15]. HCV antibodies develop in response to HCV infection and persist for life. In the absence of clinical data, the possibility of previous drug therapy cannot be excluded. The possibility of an initial false positive serology result may not be completely ruled out. In Low- and Middle-Income Countries, though Rapid Diagnostic Tests (RDTs) are widely used for HCV screening, only a few have received CE-IVD marking and only two have been pre-qualified by WHO. Hence, results generated by RDTs not validated are unreliable. It is therefore imperative that validated quality assured laboratory tests kits are utilized for anti-HCV screening. In order to achieve that, the Center for Human Virology and Genomics at the Nigerian Institute of Medical Research which is now listed as a WHO pre-qualification laboratory has evaluated thirteen RDTs in collaboration with the Foundation for Innovative New Diagnostics and two other countries. Similarly, the center is also collaborating with Clinton Health Access Initiative to evaluate HCV RDTs in use in the country.

In Nigeria, the older DAA combination with or without pegylated interferons are still in use although there is increasing access to the newer DAAs, hence the importance of knowing the VL and genotyping results. This study showed that the median VL was 407,430 IU/mL and more subjects (60.9%) had lower VL values between 15 - ≤800,000 IU/mL, which is an indication that they are more likely to respond well to the available antiviral therapy [16-18], compared to 39.1% of subjects with higher VL values of ≥800,000 IU/mL. Subjects with higher VL values have been reported to have lesser sustained virological response on treatment when compared with those with lower values [11-13]. It is also important to note that aside from higher VLs, other factors such as genotype 1 and male gender had been related with failure to peg-interferon and ribavirin [19,20]. With the introduction of the newer DAAs, the rate of VL decline does not correlate with sustained virological response and multiple VL assessments are no longer relevant [10]. Hence, these new agents are more ideal for patient management in this environment as this will reduce the frequency of laboratory monitoring while on treatment thereby reducing associated costs. Though, genotypes 1 (63.2%) and 4 (16.8%) have the highest prevalence in this study which previously would require longer treatment duration, the use of pangenotypic antiviral agents no longer requires determining the genotype prior to treatment. Hence, the needs for increased access to these antivirals in the country to further reduce cost and improve patient management. Although at least two VL results are required in the algorithm for diagnosis, treatment and monitoring of chronic HCV infection [10], only 6.6% of the subjects had repeat testing. It is possible that the patients did not commence treatment after the initial VL testing however, the challenge with affordability of the test in Nigeria had been an issue. The introduction of the pangenotypic antiviral agents will indeed increase access to treatment especially that these drugs amongst other benefits improves cure rates, that is, sustained virological response >90% and is active against all genotypes. The need to determine genotype will no longer be required thereby cutting down the cost of managing HCV infections.

The results of genotyping showed that 29.1% of subjects had undetectable genotyping results and it was also confirmed that in those subjects, their VL was equally undetectable. Since we have a high rate of subjects with cleared HCV infection despite positive antibody results, it is suggested that if required, HCV genotype test is ordered only after a detectable VL result is available in circumstances where the newer DAAs are not available. During the four years of testing under review, there were more males accessing treatment probably because the prevalence of HCV is higher in males than females. It was also observed that subjects above 41years of age had higher prevalence of detectable VL. Therefore, facility-based testing for HCV should focus on these subjects, particularly those of the male gender because it will increase case identification and linkage to care which will be more cost-effective [10]. There is therefore a need to improve access to HCV treatment to ensure the elimination of the infection by year 2030. This study showed that there was no significant association between VL and genotype. Similar findings have also been reported in a multi-country study where median VLs did not significantly vary among different genotypes [21]. The median VL values were not associated with any gender across the genotypes in this study; nonetheless, studies have also shown that subjects with lower VL tend to achieve sustained virological response (SVR) much better than those with higher values [11-13]. Some studies have observed that women tend to have better chances of SVR [22]. Nevertheless, the recent generations of pangenotypic antiviral therapies have realized higher SVR and the prognostic factors such as sex, VL and genotype may not have the same importance and strength as before.

**Limitation:** the subjects from whom data were abstracted for review were not true representative of the population in the country and some subjects did not have repeat test results as required for effective management. This could have been due to the high cost of testing therefore, selecting for those who could afford the tests.

## Conclusion

Genotypes 1 and 4 have the highest prevalence, and a greater proportion of subjects had VL values ≤800,000 IU/mL, which is an indication that they are more likely to respond well to the available antiviral therapy. Therefore, access to these antivirals will greatly improve management of HCV infection in Nigeria.

### What is known about this topic

- Nucleic acid testing technology is used for confirming HCV infection;
- Patients with high VL and certain genotypes do not respond well to interferon treatment;
- Direct-acting antivirals are effective in the treatment of HCV infection and use of pangenotypic drugs does not require the genotype determination, however, access to these drugs is limited in low- and medium-income countries.

### What this study adds

- Genotypes 1 and 4 have the highest prevalence in this study;
- This study showed 58.4% HCV viremia among subjects with anti-HCV antibodies and a median VL of 407,430 IU/mL;
- More subjects (60.9%) had lower VL values which is an indication that they are more likely to respond well to available antiviral therapy.
